# Premature Primary Tooth Loss and Oral Health-Related Quality of Life in Preschool Children

**DOI:** 10.3390/ijerph191912163

**Published:** 2022-09-26

**Authors:** Monalisa Cesarino Gomes, Matheus França Perazzo, Érick Tássio Barbosa Neves, Maria Betânia Lins Dantas Siqueira, Saul Martins Paiva, Ana Flávia Granville-Garcia

**Affiliations:** 1 Department of Dentistry, Unifacisa Centro Universitário, Campina Grande 58408-326, PB, Brazil; 2 Department of Pediatric Dentistry and Orthodontics, Federal University of Minas Gerais, Belo Horizonte 31270-901, MG, Brazil; 3 Department of Dentistry, State University of Paraíba, Campina Grande 58429-500, PB, Brazil

**Keywords:** child, preschool, tooth, deciduous, tooth loss, quality of life, oral health, social environment

## Abstract

The present study aimed to evaluate the association between premature primary tooth loss and oral health-related quality of life (OHRQoL) in preschool children. A cross-sectional study was conducted in 769 5-year-old preschool children. The children and their parents or guardians answered the Brazilian version of the Scale of Oral Health Outcomes for 5-Year-Old Children for the assessment of OHRQoL. Meanwhile, clinical examinations were performed for the assessment of premature primary tooth loss. Unadjusted and adjusted multilevel Poisson regression models were utilized to investigate the associations between the variables. In the parental version of the scale, premature posterior primary tooth loss (rate ratio [RR] = 2.65; 95% confidence interval [CI] = 1.51–4.68), weak sense of coherence (RR = 2.25; 95% CI = 1.62–3.11), and visit to a dentist (RR = 1.61; 95% CI = 1.04–2.52) were associated with OHRQoL. Based on the children’s perceptions, only the preschool type was associated with OHRQoL (RR = 1.92; 95% CI = 1.21–3.05). Premature posterior primary tooth loss had a greater impact on OHRQoL based on the parents’ perception, whereas only the preschool type was associated with OHRQoL based on the children’s perception.

## 1. Introduction

Oral health-related quality of life (OHRQoL) is described as the extent to which oral health problems can affect daily life [1]. The Scale of Oral Health Outcomes for 5-Year-Old Children (SOHO-5) is a validated questionnaire used to measure the impact of oral health conditions on the OHRQoL of 5-year-old children based on the perceptions of children themselves and their parents or guardians [2]. Although parents or guardians are the main decision-makers for children at this age, a complementary assessment of children’s perceptions favors a better understanding of the situation and can assist in the planning of adequate public health policies [2,3].

Most studies on oral health problems assess the impact of dental caries, traumatic dental injury (TDI), and malocclusion [4,5,6]. However, there is a gap in knowledge on the impact of premature primary tooth loss on OHRQoL. Dental caries is one of the main reasons for tooth loss in children, and its risk factors include poor oral hygiene, diet, and quality of oral bacterial flora [7]. Premature tooth loss can result in chewing difficulties, altered occlusion, and phonation problems and even impact dental or facial esthetics [8,9,10].

A study conducted in 8-to-9-year-old children found that premature primary molar loss influenced OHRQoL, and the prevalence of early tooth loss was 65.4% [11]. Another observational prospective cohort study was conducted in children aged 6–10 years to assess the impact of early primary molar loss on OHRQoL. This study provides evidence that early tooth loss is associated with a poor OHRQoL. Moreover, it indicates that access to dental treatment can positively impact the OHRQoL of children with dental caries and early primary molar loss. However, a questionnaire not validated for the age group was used, and deciduous molars were exclusively evaluated [12]. A qualitative study found that premature primary tooth loss can lead to functional limitations and negatively influence social interactions [13]. However, no studies have yet evaluated the impact of premature primary tooth loss in children 5 years of age.

In the present study, premature anterior and posterior primary tooth loss was analyzed considering individual and contextual determinants. The understanding of contextual factors involved in the disease process enables the identification of high-risk situations and consequently assists in the understanding of the impact of premature tooth loss on quality of life based on the perceptions of young children and their parents [14,15]. Considering this gap in knowledge, the present study aimed to assess the association between premature primary tooth loss and OHRQoL in preschool children through a multilevel analysis.

## 2. Materials and Methods

A cross-sectional study was conducted to assess the oral health status of 5-year-old children and determine its impact on OHRQoL. The study was conducted at public and private preschools between August and December 2015 in the city of Campina Grande, which is a medium-size city located in northeast Brazil.

The study population was selected using a probabilistic cluster method for complex sampling in two stages (preschools and children). A total of 263 preschools (129 public and 134 private) were registered with the Ministry of Education. Preschools were randomly selected proportional to the total number in each of the six administrative districts of the city (5 preschools in District I, 10 in District II, 5 in District III, 11 in District IV, 10 in District V, and 7 in District VI), leading to the inclusion of 28 public and 20 private preschools. The municipalities were divided by the Municipal Health Department into administrative districts for the organization of the distribution of health services. Each health district must be able to solve problems and meet all the health needs of the district population on the three levels of health care. In the second stage of the selection process, 5-year-old children enrolled at the previously selected preschools were randomly selected using a simple lottery system considering the proportion of the number of students at each preschool.

The sample size was calculated considering a 5% margin of error, 95% confidence interval (CI), and 1.6 design effect owing to the change in the precision of the estimates as a result of the two-stage sampling process. The prevalence of the impact on OHRQoL was set at 50% to maximize the sample size. Based on these parameters, the minimum sample was calculated to be 615 children and 20% was then added to compensate for possible losses, leading to a sample size of 769 5-year-old children.

### 2.1. Eligibility Criteria

Children of 5 years of age enrolled at public and private preschools were included. The children must have had no systemic impairment based on the report of a parent or guardian (systemic syndromes, physical disabilities, and learning or cognitive impairments), been in the primary dentition phase, and had no history of orthodontic treatment. Meanwhile, the included parent or guardian must have spent at least 12 h per day with the child.

### 2.2. Training and Calibration Exercises

Two researchers collected the data, and a specialist in the field served as the reference person. The images showing the oral conditions were evaluated to adjust the analysis (dental caries, TDI, and malocclusion), and questions were discussed as a group. For clinical training, 40 children were randomly selected from a preschool that did not participate in the main study and were examined on two occasions. During the first examination, the inter-examiner agreement was determined between the researchers and experienced specialist using the Kappa statistic (K = 0.89–0.90 for dental caries; K = 0.88–0.90 for TDI; and K = 0.86–0.91 for malocclusion). After 7 days, the same children were examined to determine the intra-examiner agreement (K = 0.87–1.00 for dental caries; K = 0.82–0.87 for TDI; and K = 0.94–1.00 for malocclusion). The Kappa coefficients demonstrated very good reliability for the clinical examinations (coefficients between 0.61 and 0.80 were considered good, while those between 0.81 and 1.00 were considered very good) [16].

### 2.3. Pilot Study

A pilot study was conducted prior to the data collection in the main study to determine the applicability of the instruments and dynamics of the clinical examinations. In this step, the entire proposed method was applied to 45 children from 2 schools (one public and one private) that did not participate in the main study. This step revealed no need to alter the methods.

### 2.4. Data Collection

Data were collected at the previously selected preschools. Contact was first made with the directors and coordinators of each preschool to explain the study and dynamics of data collection. Parents or guardians were then asked to participate in a meeting at their child’s preschool for clarifications regarding the objectives of the study and the obtainment of signed informed consent forms authorizing the participation of the children. On the same occasion, the parents or guardians were instructed to complete the questionnaires used for data collection. The questionnaires addressed sociodemographic characteristics, data related to the oral health of the children, and psychological aspects. After all questionnaires were completed and returned, the children were examined to assess their oral health status.

*Individual sociodemographic and oral health-related variables*. Sociodemographic data were collected using the questionnaire answered by the parents or guardians. The variables collected for the statistical analysis were the child’s sex (female or male), mother’s schooling (≤8 or >8 years of study), and family income (continuous variable). An additional question addressed whether the children had visited a dentist at least once in their life (regardless of the reason), and the answer was categorized as yes or no.

*OHRQoL.* Quality of life was assessed using the SOHO-5 [2,17]. The child version has seven items addressing difficulty eating, difficulty speaking, difficulty playing, difficulty sleeping, the avoidance of smiling owing to pain, the avoidance of smiling owing to appearance, and difficulty drinking. The response options are scored on a three-point scale (no = 0; a little = 1; a lot = 2). This version has a self-explanatory drawing for each type of answer. The parental version also has seven items that address difficulty eating, difficulty speaking, difficulty playing, difficulty sleeping, the avoidance of smiling owing to pain, the avoidance of smiling owing to appearance, and the impact of teeth on the child’s self-confidence. This version has five scored response options (not at all = 0; a little = 1; more or less = 2; a lot = 3; a great deal = 4). The questionnaire does not have domains. The final score is obtained by summing the answers to all questions. The score ranges from 0 to 14 points in the child version and from 0 to 28 points in the parental version. A higher total score denotes a greater impact on OHRQoL.

*Sense of coherence (SOC).* The SOC of the parents or guardians was measured using the version of the Sense of Coherence Scale validated for mothers of preschool children [18]. This questionnaire has 13 items, each with 5 response options addressing the components of the SOC. The total score ranges from 13 to 69 points, with higher scores denoting a stronger SOC and greater capacity to adapt to stressful situations. In the present study, the score was dichotomized by the median for the statistical analysis. Scores below and above the median were considered indicative of weak and strong SOC, respectively.

*Locus of control.* The locus of control of the parents or guardians was assessed using the Multidimensional Health Locus of Control Scale [19], which has 18 items and 3 subdivisions (internal, external, and chance). The summed score of the responses in each subscale ranges from 6 to 30 points, with a higher score denoting a lower degree of each factor. The locus of control was divided into internal when the total score was lower in the subscale of internal factors and external when the total score was lower in the subscale of external and chance factors.

*Clinical examination.* Prior to the examination, the children performed supervised brushing using an oral hygiene kit (toothbrush, toothpaste, and dental floss) provided by the researchers. The researchers used personal protective equipment (gloves, masks, caps, and whitecoats) and a head lamp (Petzl Zoom, Petzl America, Clearfield, UT, USA). The examinations were performed with the aid of a sterile mouth mirror (PRISMA, São Paulo, SP, Brazil), sterile Williams probe (WHO-621; Trinity, Campo Mourão, PR, Brazil), and gauze to dry the teeth.

Dental caries was diagnosed using the International Caries Detection and Assessment System II, the score of which ranges from 0 (sound) to 6 points (extensive cavity in dentin). Since the teeth were dried with gauze rather than compressed air, code 1 was not considered in the present study. Code 2 was recorded when early lesions were detected and codes 3 and higher when there was increasing caries severity [20]. TDI was assessed clinically based on the presence of trauma or discoloration [21] and diagnosed as follows: enamel fracture, enamel or dentin fracture, complicated crown fracture, extrusive luxation, lateral luxation, intrusive luxation, and avulsion. Tooth discoloration due to associated trauma was also considered. TDI was dichotomized as present or absent. Malocclusion was recorded by type using the criteria proposed by Grabowski et al. [22]. The following malocclusions were investigated: increased overbite (>2 mm), increased overjet (>2 mm), anterior open bite, anterior crossbite, and posterior crossbite.

The response for premature primary tooth loss was recorded as “yes” when a child was missing some primary tooth at the time of the examination or “no” when the child had all primary teeth at the time of the examination considering the chronology of the eruption or exfoliation of primary teeth. In cases of doubt (anodontia), the parent or guardian was asked whether the child had lost any primary teeth. Primary tooth loss was considered premature when it occurred at least 1 year prior to normal exfoliation or after radiographic proof that the permanent successor was still in Nolla stage six (complete crown formation and root formation initiated) [23]. As the approximate age at which the first primary teeth exfoliate (lower central incisors) is 6 years, a missing tooth prior to this period implies premature loss [24]. Since the present investigation was a school-based study, only clinical evaluations were performed (no radiographic examinations).

After the clinical examinations, a fluoride varnish (Duraphat^®^; 5% NaF) was applied to the children’s teeth. The researchers also wrote a letter to the parents or caregivers explaining the oral health status of their child.

*Contextual variables.* The following four variables were investigated to evaluate contextual influences on OHRQoL: type of preschool (public or private), average income of the neighborhood in which the preschool was located, quantity of family health teams, and quantity of oral health teams in the administrative district in which the preschool was located. Information on neighborhood income was obtained from data collected by the Brazilian Institute of Geography and Statistics in the city. Data on the quantity of family health teams and oral health teams in the districts were collected from the municipal health secretary and data on the type of school from the schools during the first visit.

### 2.5. Statistical Analysis

The data were organized and statistically analyzed using the Statistical Package for the Social Sciences for Windows (version 22.0; IBM Inc., Armonk, NY, USA). Unadjusted and adjusted multilevel Poisson regression models were utilized to describe the associations between the predictors and outcome. The response variable was the impact on the OHRQoL of the preschool children. The scores on the OHRQOL questionnaire (0 to 14 points in the child version and 0 to 28 points in the parental version) were considered continuous variables in the statistical analysis. The multilevel Poisson regression analysis involved a fixed effects model with random intercepts to assess the associations between the dependent and independent variables (individual and contextual). This strategy enabled the estimation of the rate ratios (RRs) between the comparison groups and the respective 95% CIs.

In the first step, a null model was used to determine the variability of the data prior to considering the individual and contextual characteristics. In model 2, individual covariables were added. Model 3 included individual and contextual covariables. For the selection of variables, individual variables with a p-value of <0.20 in the bivariate multilevel Poisson regression analysis were incorporated, and those with a p-value of <0.05 were maintained in model 2. Next, contextual variables with a p-value of <0.20 in the bivariate multilevel Poisson regression analysis were incorporated, and those with a p-value of <0.05 were maintained in model 3 (final model). The final model was adjusted for dental caries, TDI, and malocclusion. The goodness of fit of the models was calculated based on deviance values (−2 log likelihood), and the significance of the models was determined using the likelihood ratio test.

### 2.6. Ethical Aspects

This study was approved by the institutional review board of *Universidade Estadual da Paraíba* (certificate number: 38937714.0.0000.5187) and conducted in accordance with the Declaration of Helsinki as well as Resolution 466/2012 of the National Board of Health. All parents or guardians signed an informed consent form prior to the data collection. All students received information on the study protocol and agreed to participate.

## 3. Results

The final study sample consisted of 769 pairs of children and parents or guardians. Premature primary tooth loss was diagnosed in 29.8% of the children. The sample characteristics are described in Table 1.

In the bivariate analysis, mother’s schooling, family income, history of toothache, SOC, locus of control, and type of preschool were associated with a negative impact on OHRQoL based on the perception of the children (*p* < 0.05). Meanwhile, mother’s schooling, family income, history of toothache, SOC, locus of control, premature primary tooth loss, and type of preschool were associated with a negative impact on OHRQoL based on the perception of the parents or guardians (*p* < 0.05) (Table 2).

Table 3 displays the results of the multivariate multilevel Poisson regression analyses for the parental and child versions of the SOHO-5. Based on the parents’ or guardians’ perception, history of visit to a dentist (RR = 1.61; 95% CI = 1.03–2.51), weak SOC of the parents or guardians (RR = 2.25; 95% CI = 1.63–3.11), and premature primary tooth loss (RR = 2.66; 95% CI = 1.51–4.68) were identified as individual determinants of the OHRQoL of the children after adjusting for the individual variables (model 2). After the incorporation of the contextual variables (model 3), these same variables remained associated with OHRQoL (*p* < 0.05), while no contextual determinant was associated with such. Based on the children’s perception, no individual variables were associated with a negative impact on OHRQoL. However, when the contextual determinants were incorporated, attending a public preschool was found to be associated with a greater negative impact on OHRQoL (RR = 1.92; 95% CI = 1.21–3.05).

Adjustment variables: dental caries, traumatic dental injury, and malocclusion.

Individual variables incorporated into model 2 for the analysis of the scores in the parental version include: mother’s schooling, monthly family income, premature posterior tooth loss, sense of coherence, locus of control, visit to a dentist, dental caries, traumatic dental injury, and malocclusion.

Contextual variables incorporated into model 3 for the analysis of the scores in the parental version include: type of preschool, income of the neighborhood, and number of oral health teams.

Individual variables incorporated into model 2 for the analysis of the scores in the child version include: mother’s schooling, monthly family income, sense of coherence, locus of control, visit to a dentist, premature anterior tooth loss, dental caries, traumatic dental injury, and malocclusion.

## 4. Discussion

The present results demonstrate the importance of considering the perceptions of both parents or guardians and children in the evaluation of OHRQoL. Although the care of children is under the direct responsibility of their parents or guardians and dependence is greater at this age, the reports of children are also important and should complement the decision-making process in health-related matters [2,25].

To the best of our knowledge, this is the first study to assess the association between premature primary tooth loss and OHRQoL in preschool children through an analysis of individual and contextual determinants. Premature posterior primary tooth loss was associated with a greater impact on the OHRQoL of the children based on the perception of their parents or guardians. However, this variable did not affect OHRQoL based on the perception of the children, confirming the importance of a broader assessment of OHRQoL when evaluating children. Moreover, premature anterior primary tooth loss was not associated with OHRQoL in either version of the SOHO-5. Previous studies investigating children 6–9 years of age found that premature primary molar loss negatively affected OHRQoL [11,12]. However, these studies were conducted in a different age group and utilized methods that differ from our approach. The present study evaluated the early loss of all deciduous teeth and conducted a multilevel assessment, considering that individuals are part of a context that influences life and health conditions.

Premature primary tooth loss is mainly the result of two conditions—dental caries and TDI [26,27]. In the present study, the prevalence of avulsion (tooth loss due to trauma) was low (1.8%). Therefore, premature tooth loss in preschool children may be more associated with toothache and a greater severity of dental caries. Studies have shown that caries is a highly prevalent multifactorial condition among preschool children [28,29], one of the main consequences of which is premature primary tooth loss [30,31]. This underscores the need for a comprehensive evaluation of oral health. Thus, emphasis should be placed on educating families on home oral hygiene and the use of essential aids for reducing the incidence of primary tooth loss, including products based on biomimetic hydroxyapatite [7,32,33].

The impact of premature posterior primary tooth loss on OHRQoL perceived by the parents or guardians was the same after the multivariate analysis, demonstrating that the impact on OHRQoL is independent of the context in which the child is inserted. This finding is compatible with the association between a history of visit to a dentist and a greater impact on OHRQoL perceived by the parents or guardians. Indeed, studies have shown that the main reason for visiting a dentist among preschool children is curative treatment for toothache [34,35], which may be related to a greater impact on OHRQoL.

A weak SOC of the parents or guardians was associated with a greater impact on the OHRQoL of the children in the parental version of the SOHO-5. The SOC of parents or guardians is a psychosocial parameter for understanding the oral health and behavior of children [36]. Herein, this association was found in the parental version only, suggesting possible parental guilt over the oral health problems of their children, as confirmed in previous studies [37,38].

Based on the perception of the children, only one contextual variable (type of preschool) was associated with OHRQoL, demonstrating the importance that should be given to the context in which a child is inserted independently of individual characteristics. In the present investigation, studying at a public preschool was associated with a greater impact on OHRQoL. A previous study has found that children in an unfavorable social context also had worse OHRQoL based on the reports of parents or guardians [39]. Thus, the school should be considered an important factor when planning oral health promotion measures, as it is where children spend a considerable part of their time. The school setting is important to the intellectual development of children and influences health behaviors. In Brazil, most children who study at public schools are from underprivileged families who live in areas of social deprivation [40].

The findings suggest a possible lack of importance given to the primary dentition by parents or guardians, resulting in the need for extractions. Further, children may not remember the episode leading to premature tooth loss and may not perceive the current impact on chewing function and esthetics. At the preschool age, esthetic issues may not influence OHRQoL. Thus, longitudinal studies should be conducted to better evaluate the influence of premature primary tooth loss on OHRQoL.

Considering the cross-sectional design of the present investigation, further studies are needed to clarify the cause-and-effect relationship of the variables analyzed. Moreover, as this was a school-based study, no radiographic examinations were performed to determine the Nolla stage. Thus, measurement bias might have occurred. Nonetheless, this is the first study to analyze the influence of premature anterior and posterior primary tooth loss on the OHRQoL of 5-year-old children through the perceptions of parents or guardians and children themselves through a contextual analysis. The association between premature primary tooth loss and OHRQoL in 5-year-old children was controlled for dental caries, TDI, and malocclusion. Therefore, the impact encountered was independent of other oral health conditions. This finding underscores the importance of adequate planning of public policies directed at the oral health of children to avoid the occurrence and progression of oral health problems that could result in tooth loss.

## 5. Conclusions

Based on the perception of parents or guardians, premature posterior primary tooth loss, visit to a dentist, and weak SOC of parents or guardians greatly influence the OHRQoL of their children. Based on the perception of children, only the type of preschool is associated with OHRQoL.

## Figures and Tables

**Table 1:** Individual and contextual characteristics of the sample.

Variables	*n* (%)/Mean (SD)
Individual characteristics	
Child’s sex	
Female	403 (52.4)
Male	366 (47.6)
Mother’s schooling	
≤8 years of study	230 (30.0)
>8 years of study	536 (70.0)
Monthly family income	1877.05 (2595.29)
Visit to a dentist	
Yes	334 (43.5)
No	434 (56.5)
Sense of coherence	
Weak	321 (41.7)
Strong	448 (58.3)
Locus of control	
Internal	520 (68.1)
External	244 (31.9)
Premature primary tooth loss	
No	540 (70.2)
Yes	229 (29.8)
Contextual characteristics	
Type of preschool	
Public	298 (38.8)
Private	471 (61.2)
Number of family health teams in the neighborhood of the preschool (mean)	17.53 (4.59)
Number of oral health teams in the neighborhood of the preschool (mean)	8.94 (2.13)
Income of the neighborhood of the preschool (mean)	1026.64 (474.21)

**Table 2:** Bivariate analysis of the association between the general SOHO-5 scores and individual and contextual characteristics.

	SOHO-5 Parental Version			SOHO-5 Child Version		
Variable	Mean (SD)	*p*-Value	RR (95% CI)	Mean (SD)	*p*-Value	RR (95% CI)
Individual characteristics						
Sex						
Female	1.16 (2.74)	0.30	1.23 (0.82–1.85)	2.20 (3.22)		1.00
Male	1.00 (2.56)		1.00	2.53 (3.45)	0.46	1.10 (0.83–1.46)
Mother’s schooling						
≤8 years of study	1.94 (3.86)	<0.001	2.72 (1.84–4.04)	3.53 (4.03)	<0.001	1.89 (1.44–2.49)
>8 years of study	0.70 (1.80)		1.00	1.88 (2.88)		1.00
Monthly family income	-	<0.001	1.00 (0.99–1.00)	-	<0.001	1.00 (1.00–1.01)
Visit to a dentist						
Yes	1.34 (2.99)	0.14	1.34 (0.89–2.01)	2.25 (3.22)	0.08	1.26 (0.96–1.64)
No	0.88 (2.35)		1.00	2.47 (3.45)		1.00
Sense of coherence						
Weak	1.63 (3.41)	<0.001	2.69 (1.87–3.87)	2.76 (3.56)	0.01	1.42 (1.08–1.87)
Strong	0.68 (1.84)		1.00	2.10 (3.16)		1.00
Locus of control						
Internal	0.91 (2.27)		1.00	2.21 (3.19)		1.00
External	1.36 (3.09)	0.02	1.59 (1.06–2.38)	2.70 (3.63)	0.006	1.50 (1.12–2.01)
Premature anterior tooth loss						
No	0.99 (2.58)		1.00	2.48 (3.46)		1.00
Yes	1.28 (2.81)	0.47	1.16 (0.77–1.76)	2.05 (2.94)	0.07	0.77 (0.57–1.02)
Premature posterior tooth loss						
No	0.99 (2.50)		1.00	2.35 (3.35)		1.00
Yes	4.32 (5.19)	<0.001	5.40 (3.17–9.17)	2.79 (2.12)	0.33	1.19 (0.83–1.70)
Contextual characteristics						
Type of preschool						
Public	1.80 (3.70)	<0.001	3.18 (2.23–4.54)	3.54 (4.05)	<0.001	2.50 (1.97–3.17)
Private	0.62 (1.52)		1.00	1.63 (2.56)		1.00
Income of the neighborhood	-	0.07	0.99 (0.99–1.00)	-	0.72	1.00 (0.99–1.01)
Number of oral health teams	-	0.19	0.93 (0.85–1.03)	-	0.10	0.95 (0.90–1.01)
Number of family health teams	-	0.29	1.01 (0.98–1.05)	-	0.06	0.97 (0.95–1.01)

**Table 3:** Multilevel Poisson regression analysis of the associations between the SOHO-5 (parental and child versions) scores and individual and contextual variables.

	SOHO-5 Parental Version			SOHO-5 Child Version		
	Model 1 (“Null”)	Model 2	Model 3	Model 1 (“Null”)	Model 2	Model 3
Fixed effects		RR (95% CI)			RR (95% CI)	
Intercept	0.71 (0.52–0.98)	0.57 (0.03–11.87)	0.15 (0.07–0.35)	2.05 (1.65–2.55)	0.55 (0.15–2.04)	1.29 (0.69–2.39)
Individual variables						
Premature posterior tooth loss						
No		1.00	1.00		-	-
Yes		2.66 (1.51–4.68)	2.65 (1.51–4.68)		-	-
Sense of coherence of the parents or guardians						
Strong		1.00	1.00		-	-
Weak		2.25 (1.63–3.11)	2.25 (1.62–3.11)		-	-
Visit to a dentist						
Yes		1.61 (1.03–2.51)	1.61 (1.04–2.52)		-	-
No		1.00	1.00		-	-
Contextual variables						
Type of preschool						
Public			-			1.92 (1.21–3.05)
Private			-			1.00
Random effects						
Deviance (–2 log likelihood)	51,083.858	38,724.023	38,712.279	47,838.025	43,590.761	43,585.339

Model 1 (“null”): unconditional model; Model 2: with individual variables; Model 3: with individual and contextual variables.
